# Protective effect of Xin Mai Jia ultrafiltration extract on human umbilical vein endothelial cell injury induced by hydrogen peroxide and the effect on the NO-cGMP signaling pathway

**DOI:** 10.3892/etm.2014.1700

**Published:** 2014-05-02

**Authors:** YALING YIN, JIA WAN, PENG LI, YANLONG JIA, RUILI SUN, GUOPIN PAN, GUANGRUI WAN

**Affiliations:** 1 School of Basic Medical Sciences, Xinxiang Medical University, Xinxiang, Henan 453003, P.R. China; 2 Department of Nephrology, The First Affiliated Hospital, Zhengzhou University, Zhengzhou, Henan 450052, P.R. China; 3 College of Pharmacy, Xinxiang Medical University, Xinxiang, Henan 453003, P.R. China; 4 Department of Inspection, Xinxiang Medical University, Xinxiang, Henan 453003, P.R. China; 5 Modern Technology Education Center, Xinxiang Medical University, Xinxiang, Henan 453003, P.R. China

**Keywords:** Xin Mai Jia, human umbilical vein endothelial cell, hydrogen peroxide, atherosclerosis

## Abstract

The aim of the present study was to evaluate the protective effect of the ultrafiltration extract of Xin Mai Jia (XMJ) on a human umbilical vein endothelial cell (HUVEC) injury model induced by hydrogen peroxide (H_2_O_2_), by providing experimental data to investigate the mechanism and efficacy underlying the therapeutic effects on atherosclerosis. HUVECs were first injured by H_2_O_2_ and then varying final concentrations of the Chinese herb extract were added. Effects of the XMJ extract on morphology, activity, monolayer permeability, biochemical indicators, cytokines, endothelial nitric oxide synthase (eNOS) protein content and eNOS gene expression in the HUVECs were analyzed. H_2_O_2_ significantly promoted HUVEC injury. The XMJ ultrafiltration extract significantly improved the morphological changes in the injured HUVECs. In addition, XMJ treatment increased cell activity and decreased monolayer permeability. The expression levels of intracellular adhesion molecule-1, vascular adhesion molecule-1, interleukin-1 and -6 and nuclear factor-κB decreased, while the expression levels of matrix metalloproteinase-2 and tissue inhibitor of metalloproteinase-2 increased with XMJ administration. Increased levels of nitric oxide (NO), eNOS protein and eNOS gene expression were also observed. Therefore, the XMJ ultrafiltration extract exhibits marked anti-inflammatory effects and antioxidant abilities. These properties significantly inhibited the H_2_O_2_-induced injury of HUVECs, which may be associated with the NO-cyclic guanosine monophosphate signaling pathway.

## Introduction

Vascular endothelial cells, the natural barrier between the blood and tissue, secrete various vasoactive substances, cytokines and neurotransmitters, including nitric oxide (NO), interleukins (ILs), adhesion molecules, endothelin and tissue factors (1–3). These substances participate in and influence the organic movement of substances, blood coagulation, immune responses and other essential activities. Previous studies have shown that oxidative stress injury in vascular endothelial cells is the cause of various vascular diseases, including atherosclerosis (AS), diabetes and hypertension (4,5). Oxidative stress generates reactive oxygen species (ROS), such as the superoxide anion (^•^O^2−^), the hydroxyl radical (^•^OH) and hydrogen peroxide (H_2_O_2_). ROS bind to the nuclear receptor of vascular endothelial and smooth muscle cells as a ligand, or directly regulate gene expression as signaling molecules. These functions enhance the adhesion and migration of monocytes to the tunica intima, which is important during the development of AS (6,7).

NO is a neurotransmitter secreted by vascular endothelial cells, which can inhibit monocyte-macrophage and platelet adhesion, decrease the monolayer permeability of vascular endothelial cells and reduce vascular endothelial cell and smooth muscle cell proliferation (8,9). Previous studies have shown that 30% of NO in the blood is derived from endothelial nitric oxide synthase (eNOS) genes expressed by vascular endothelial cells. NO diffuses to nearby endothelial and smooth muscle cells, activates soluble guanosine monophosphate cyclase within the cytoplasm, decomposes guanosine triphosphate and generates cyclic guanosine monophosphate (cGMP), which results in biological effects, including vasodilation, that play an important role in AS (10).

Xin Mai Jia (XMJ) is a traditional Chinese medicine (TCM) used in the treatment of AS that can effectively inhibit AS occurrence and development (11). XMJ is prepared using the following raw materials based on weight: Functional red kojic rice powder, 10–35%; kudzu flavonoid powder, 1–10%; soybean isoflavone powder, 1–8%; bamboo leaf flavone powder, 1–8%; resveratrol powder, 1–8%; hawthorn powder, 1–6%; *Gastrodia* powder, 1–6%; *Auricularia auricula* powder, 1–30%; hippocampus powder, 0.1–0.2%; astaxanthin powder, 0.008–0.04%; menthol powder, 0.1–0.3%; and resistant starch, 20–50%. To investigate the inhibitory mechanism of XMJ on the occurrence and development of AS, the present study evaluated the effects of intervention with the ultrafiltration extract from XMJ on a human umbilical vein endothelial cell (HUVEC) injury model induced by H_2_O_2_. The effect on the NO-cGMP signaling pathway, caused by the inhibition of the H_2_O_2_-induced injury, was also investigated.

## Materials and methods

### Drugs and chemicals

XMJ crude drugs were purchased from Beijing Tong Ren Tang Co., Ltd. (Beijing, China). Phospho-eNOS-3 antibodies were purchased from Beijing Boaosen Biotechnology Co., Ltd. (BS-3447R; Beijing, China), horseradish peroxidase-labeled goat anti-rabbit IgG (heavy and light chains) antibody was purchased from Beijing Zhongshan Golden Bridge Biotechnology Co., Ltd. (PV-9003; Beijing, China) and Cy3-labeled goat anti-rabbit IgG (heavy and light chain) antibodies were purchased from Biyuntian Institute of Biotechnology (P0183; China). Lovastatin, H_2_O_2_, diethyl carbonate, XTT, phenazine methosulfate (PMS), diethylpyrocarbonate and avian myeloblastosis virus were purchased from Sigma-Aldrich (St. Louis, MO, USA). Zhibituo was purchased from Chengdu Diao Jiuhong Pharmaceutical Factory (Chengdu, China). Hematoxylin and eosin (HE) and Coomassie Brilliant Blue stains were purchased from Changsha Lixin Biotechnology Co., Ltd. (Changsha, China). Kits for the detection of malondialdehyde (MDA), superoxide dismutase (SOD), NO, interleukin (IL)-1, IL-6, intracellular adhesion molecule (ICAM)-1, vascular adhesion molecule (VCAM)-1, matrix metalloproteinase (MMP)-2, tissue inhibitor of metalloproteinase (TIMP)-2 and nuclear factor (NF)-κB were purchased from R&D Systems (Minneapolis, MN, USA). Other reagents were analytically pure and made in China.

### Cell experiment protocol

HUVECs were obtained from HUVEC cell lines purchased from Acti Corp. (Irvine, CA, USA) and were routinely maintained in phenol-red containing Dulbecco’s modified Eagle’s medium (Gibco Construction, LLC, Cleveland, TN, USA), which was supplemented with 15% newborn calf serum, 100 U/ml penicillin and 0.1 mg/ml phytomycin, in a 37°C incubator in an atmosphere of 5% CO_2_. The third generation of HUVECs were used in the study. The cells were randomly divided into eight groups and incubated for 24 h with the corresponding drugs. The first group of cells were incubated in Kreb’s solution and were classified as the blank control group (n=6). The second group of cells were incubated in 500 mg/l XMJ and were classified as the XMJ control group (n=6). The third group of cells were incubated in 200 μmol/l H_2_O_2_ and were classified as the model group (n=6). The fourth group of cells were incubated in 1 μmol/l lovastatin and 200 μmol/l H_2_O_2_ and were classified as the lovastatin group (n=6). The fifth group of cells were incubated in 50 μmol/l zhibituo and 200 μmol/l H_2_O_2_ and were classified as the zhibituo group (n=6). The sixth group of cells were incubated in 25 μmol/l XMJ and 200 μmol/l H_2_O_2_ and were classified as the low-dose XMJ group (n=6). The seventh group of cells were incubated in 50 μmol/l XMJ and 200 μmol/l H_2_O_2_ and were classified as the middle-dose XMJ group (n=6). Finally, the eighth group of cells were incubated in 50 μmol/l XMJ and 200 μmol/l H_2_O_2_ and were classified as the high-dose XMJ group (n=6). Following the individual treatments, the cultured cells were obtained for the subsequent tests.

### Ultrafiltration membrane extract preparation for XMJ

In total, 1,000 g XMJ crude drugs were placed in a container filled with 6,000 ml water, which was heated in a microwave oven for 1 h at 1,000 W. A four-gauze filter was used to obtain one type of liquid medicine. Next, 6,000 ml water was added to the container again prior to repeating the aforementioned procedure. Following the production of two types of liquid medicine, the liquid was filtered with sterile absorbent cotton. Ultrafiltration technology of refining XMJ was then used for water decoction under 0.5 kPa/m^3^ at 25°C and 100 l/h/m^2^. The 5,000 ml filtrate was condensed to 1,000 ml, which was equivalent to 1 ml liquid medicine containing 1 g XMJ. Finally, the mother liquor was labeled and stored in a refrigerator at 4°C prior to use.

### HUVEC staining

Cell growth on the wall of the bottle was observed in each experimental group. The supernatant in the 96-well plate was discarded and the cells were washed three times with phosphate-buffered saline (PBS; pH 7.4) for 1 min for each repetition. Next, the cells were soaked in 95% ethanol for 20 min and washed with PBS twice at 1 min for each repetition. Hematoxylin was used to dye the cells for 2–3 min and then the cells were washed with pure water. The cells were observed under a microscope (Olympus Corp., Tokyo, Japan). For deeply-stained nuclei, a 1% solution of hydrochloric acid and alcohol was used to dilute the cells into dichroic for 5 sec. The cells were washed with pure water and placed in 70% alcohol for dehydration for 10 min and then in 90% alcohol for 10 min. The cells were washed with distilled water and dyed using alcoholic eosin for 2–3 min. Finally, the cells were dried and mounted on slides using neutral gum.

### HUVEC activity detection

When the concentration of the cells growing on the walls of the bottles in each experimental group reached 10^6^ cells/cm^2^, the following steps were undertaken. The cells were washed twice with PBS, 0.25% trypsin was added and the flasks were shaken to remove the cells from the wall of the bottle with the aid of nozzle-pipe blowing. Thiophene azole blue solution (1 g/l; 37°C; volume fraction, 5%) was added to each well and 10-μl samples were obtained from each well and added to an automatic cell counting board (Countstar). The viability rate, average compactness and aggregation rate of the cells in each group were measured using a Countstar automatic cell-counting instrument.

### HUVEC monolayer permeability determination

According previous studies (12,13), the osmotic reflection coefficient (σ) and the endothelial monolayer filtration coefficient (Kf; μl/min/cm^2^/kPa) were calculated according to the following formulas: Kf = total water flux (Jv)/(ΔP−σ·Δπ); σ = 1−C_F_/C_P_; π (kPa) = C(mOsm/l) × 2.6(kPa/l/mOsm); where Jv (μl·min^−1^·cm^−2^)=V/S·min; C_P_ was the upper chamber white protein concentration; C_F_ was the inferior vena albumin concentration; C was the albumin milli seepage quantity concentration; ΔP was the perfusion pressure; and π was the colloid osmotic pressure.

### HUVEC supernatant fluid biochemical indicator detection

Cells were seeded into six-well plates at 2 ml per well in order for the samples to be treated as a group, according to the aforementioned method. Cell supernatant fluid biochemical indicators were detected. Culture fluids were obtained separately to determine the SOD, MDA and NO concentration, as described in the kits.

### ELISA

Cells were seeded into six-well plates at 2 ml per well in order for the samples to be treated as a group, according to the aforementioned method. Following the instructions on the ELISA kit, cell supernatant fluids were obtained from the wells and the optical density (OD) value of each well was measured at a wavelength of 450 nm. The acquired absorbance value of the OD was regarded as the ordinate and the concentration of the standard liquid was considered as the abscissa. A curve was constructed, from which the curve equation was calculated. By substituting the OD values of the samples into the equation of the standard curve, IL-1, IL-6, ICAM-1, VCAM-1, MMP-2, TIMP-2 and NF-κB levels were calculated.

### Western blot analysis

As previously described (14), the supernatant in the 96-well plates was discarded and the eNOS protein content of each experimental group of adhered HUVECs was assayed. Pre-cooled cell lysates were added, proteins were obtained and the protein concentration was detected using the bicinchoninic acid method.

### Immunohistochemical method

Cells were soaked in 95% ethanol for 20 min, washed with PBS twice for 1 min per wash and sealed with animal serum. An appropriate dilution (1:400) of phospho-eNOS-3-endothelial antibodies was added and the cells were incubated overnight at 4°C. Next, horseradish peroxidase-labeled goat anti-rabbit IgG antibodies were added. Mayer’s hematoxylin was used to stain the cells for a second time, and the cells were dehydrated and dried using gradient alcohol. Transparent xylene was also added and the cells were mounted on slides using neutral gum. Finally, the cells were observed under a microscope and images were obtained. The results were processed and analyzed using analysis software for OD.

### Immunofluorescence

The supernatant in the 96-well plates was discarded and 0.01 mol/l PBS (pH 7.4) was added dropwise into the specimen sheet to be tested, which was discarded after 10 min. To keep the specimen wet to a certain extent, a l:200 phospo-eNOS-3-endothelial antibody dilution was added and the cells were incubated overnight at 4°C. The membranes were washed with Tris-buffered saline Tween-20 three times and a dilution (l:l,000) of Cy3-labeled goat anti-rabbit IgG antibodies were added and used to completely cover the specimens. The specimens were placed into an enamel box with a lid and incubated for 30 min. The slides were removed from the enamel box and placed on the slide shelves. Next, the slides were washed with 0.01 mol/l PBS (pH 7.4) and soaked in three water jars containing 0.01 mol/l PBS (pH 7.4). The specimens were processed and analyzed using analysis software for OD.

### Quantitative polymerase chain reaction (qPCR)

Total cellular RNA was extracted using a TRIzol reagent kit. The primers were synthesized by Takara Biotechnology Dalian Co., Ltd. (Dalian, China) and the forward and reverse primer sequences (5′-3′) were as follows: e1, GGGACCACATAGGTGTCTGC; and e2, CCAGCACAGCTACAGTGAGG. The 10-μl reaction system was composed of 5 μl SYBR Premix *Taq* TM (2X) reaction liquid, 0.25 μl PCR forward primer (10 μM), 0.25 μl PCR reverse primer (10 μM), 0.5 μl cDNA template and 4 μl deionized water. The recorded temperatures of the melting curve ranged between 60 and 95°C. Following the reaction, the PCR samples were processed separately by agarose gel electrophoresis to verify whether the fragments had been amplified. When the qPCR reaction had been completed, the data were collected and analyzed using the computer analysis software, PikoReal Software 2.1 (Thermo Fisher Scientific, Waltham, MA, USA). The corresponding Ct value was calculated after adjusting the baseline cycle threshold according to the requirements of the software.

### Statistical analysis

All data are expressed as the mean ± standard error. One-way analysis of variance and the Student-Newman-Keuls test for multiple comparisons were used to compare the data among the various groups. Statistical analysis was performed using the SPSS 13.0 statistical software (SPSS, Inc., Chicago, IL, USA) and P<0.05 was considered to indicate a statistically significant difference.

## Results

### Protective effect of XMJ on HUVEC injury induced by H_2_O_2_ observed under a microscope

**Figure 1:**
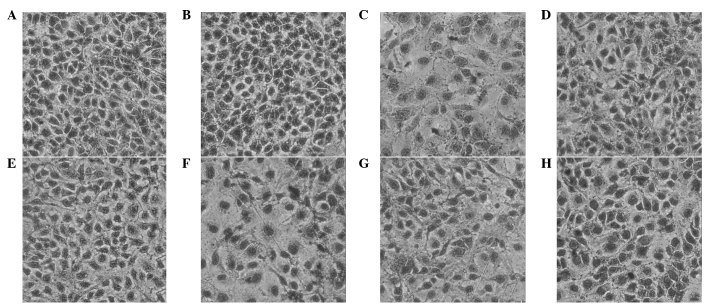
Microscopic images showing the protective effect of XMJ on HUVEC injury induced by H_2_O_2_ (magnification, ×400; HE stain) in the (A) blank control, (B) XMJ control, (C) model, (D) lovastatin, (E) zhibituo, (F) low-dose XMJ, (G) medium-dose XMJ and (H) high-dose XMJ groups. XMJ, Xin Mai Jia; HUVEC, human umbilical vein endothelial cell; HE, hematoxylin and eosin; H_2_O_2_, hydrogen peroxide.

HUVECs stained with HE were observed under a microscope (magnification, ×400) and the observations were as follows. HUVEC apoptosis was significantly reduced in the high-dose XMJ group. Cytoplasmic staining was relatively uniform and the metachromatic particles of the nuclei were not marked. The cells were arranged closely and their morphology was normal. Evident differences were identified when comparing these cells with the model group. Higher-dose XMJ demonstrated a significant protective effect on the HUVEC injury induced by H_2_O_2_. Lovastatin and zhibituo also exhibited marked protective effects, however, their protective effects with regard to morphology were relatively weaker when compared with the high-dose XMJ group. The protective effects of the low- and middle-dose XMJ groups were significantly weaker than that of the high-dose XMJ group, indicating the dependence of the protective effect on XMJ dosage (Fig. 1).

### Effect of XMJ on HUVEC activity

**Figure 2:**
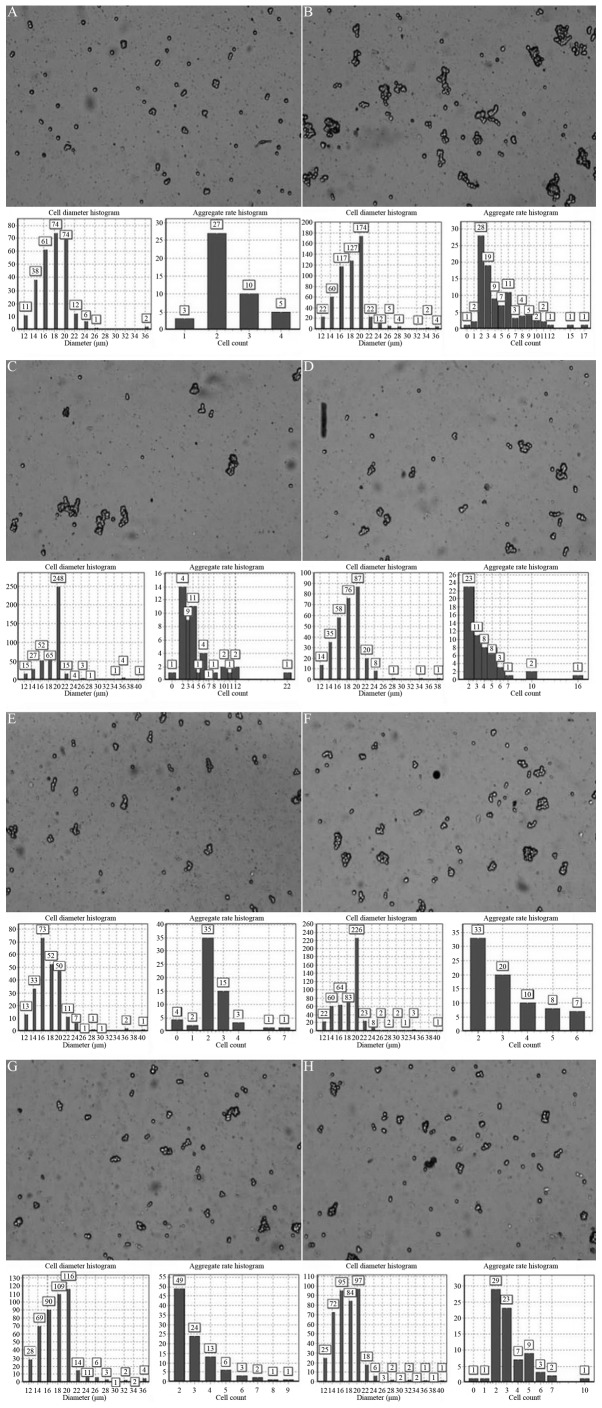
Effect of XMJ on the activity levels of HUVECs in the (A) blank control, (B) XMJ control, (C) model, (D) lovastatin, (E) zhibituo, (F) low-dose XMJ, (G) medium-dose XMJ and (H) high-dose XMJ groups, as determined by a Countstar automatic cell counter. XMJ, Xin Mai Jia; HUVECs, human umbilical vein endothelial cells; H_2_O_2_, hydrogen peroxide.

In the high-dose XMJ group, the rate of HUVEC activity was 89.54%, the average degree of compaction was 0.77 and the speed rate of polymerization was 60.83%. In the model group, the activity rate of the HUVECs was 54.13%, the average degree of compaction was 0.78 and the speed rate of polymerization was 52.52%. These results exhibited statistically significant differences (P<0.05). The significant increase in the HUVEC activity rate in the high-dose XMJ group demonstrated that a high dosage of XMJ exhibits a significant protective effect on the reduction in the activity rate of injured HUVECs induced by H_2_O_2_. Lovastatin and zhibituo also demonstrated marked protective effects on the H_2_O_2_-induced reduction in the activity rate of the HUVECs, and when compared with the model group, a significant difference (P<0.05) was observed. However, when compared with the high-dose XMJ group, the differences in the activity rate of the HUVECs in the lovastatin and zhibituo groups were not statistically significant. The protective effects of the low- and middle-dose XMJ groups on the activity rate of the HUVECs induced by H_2_O_2_ were significantly weaker than that of the high-dose XMJ group, confirming that the effect was dependent on XMJ dosage. However, the differences in the average degree of compaction and the speed rate of polymerization in the low-, middle- and high-dose XMJ groups were not statistically significant (P>0.05; Fig. 2).

### Protective effect of XMJ on the reduction of monolayer permeability in HUVEC injury induced by H_2_O_2_

In the high-dose XMJ group, the Jv of the HUVECs was 29.43±7.53 μl/min/cm^2^, the Kf was 12.43±2.24 μl/min/cm^2^/kPa and the σ was 0.59±0.08. In the model group, the Jv of the HUVECs was 44.47±8.56 μl/min/cm^2^, the Kf was 17.66±3.43 μl/min/cm^2^/kPa and the σ was 0.29±0.03. Compared with the model group, the Jv of the HUVECs in the high-dose XMJ group significantly decreased. Statistically significant differences (P<0.05) were also identified in the Kf, which markedly decreased in the high-dose XMJ group, as well as the σ, which significantly increased. These results indicated that high-dose XMJ demonstrated marked protective effects on the reduction in HUVEC monolayer permeability induced by H_2_O_2_. Lovastatin and zhibituo also exhibited evident protective effects and statistically significant differences (P<0.05) were observed when compared with the model group. However, the protective effects of high-dose XMJ were more significant than those of lovastatin and zhibituo (P<0.05). The protective effects of low- and middle-dose XMJ, based on the by H_2_O_2_-induced decrease in the permeability of the HUVEC monolayer, were significantly weaker than that of high-dose XMJ (P<0.05), thus, the protective effects were dependent on the dose of XMJ (Table I).

### Effect of XMJ on the concentration of SOD and MDA in the supernatant of HUVECs induced by H_2_O_2_

**Figure 3:**
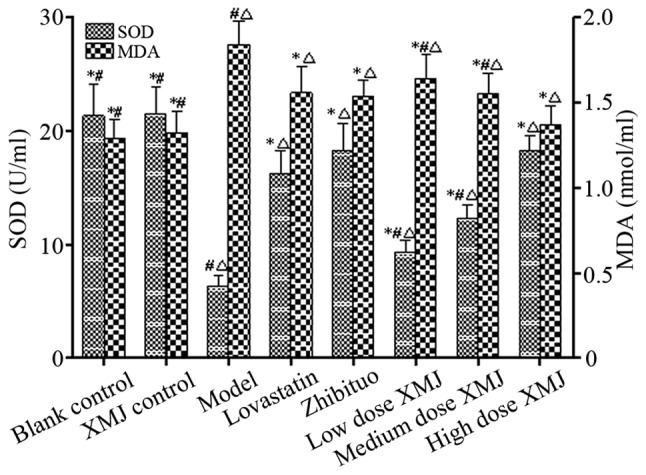
Effect of XMJ on the concentration of SOD and MDA in the supernatants of HUVECs induced by H_2_O_2_. ^*^P<0.05, vs. model group; ^#^P<0.05, vs. high-dose XMJ group; ^△^P<0.05, vs. blank control group. XMJ, Xin Mai Jia; HUVECs, human umbilical vein endothelial cells; SOD, superoxide dismutase; MDA, maldionaldehyde; H_2_O_2_, hydrogen peroxide.

In the high-dose XMJ group, the concentrations of SOD and MDA were 18.21±1.39 U/ml and 1.37±0.26 nmol/ml, respectively. In the model group, the concentration of SOD was 6.35±0.87 U/ml and the concentration of MDA was 1.84±0.26 nmol/ml. Compared with the model group, statistically significant differences (P<0.05) were observed in the high-dose XMJ group with regard to SOD concentration, which significantly increased, and MDA, which significantly decreased. Lovastatin and zhibituo also exhibited marked suppressive effects on the decrease of supernatant SOD levels and the increase of supernatant MDA levels in the HUVEC injury model induced by H_2_O_2_. The results were statistically significant (P<0.05) when compared with the model group, however, when compared with the high-dose XMJ group, the differences in HUVEC supernatant SOD and MDA concentrations in the lovastatin and zhibituo groups exhibited no statistically significant differences (P>0.05). The suppressive effects of low- and middle-dose XMJ on the decrease in supernatant SOD concentration and on the increase in supernatant MDA concentration were significantly weaker than that of high-dose XMJ (P<0.05), thus, the effects were dependent on XMJ dosage (Fig. 3).

### Effect of XMJ on the level of supernatant cytokines in the HUVEC injury model induced by H_2_O_2_

In the middle-dose XMJ group, the levels of cytokines were as follows: ICAM-1, 36.33±7.32 ng/l; VCAM-1, 36.66±1.58 μg/l; IL-1, 14.75±2.04 ng/l; IL-6, 6.05±0.84 ng/l; MMP-2, 0.671±0.07 μg/l; and TIMP-2, 2605.99±222.17 pg/l. In the model group, ICAM-1 was 71.18±6.67 ng/l, VCAM-1 was 44.81±2.09 μg/l, IL-1 was 18.34±2.14 ng/l, IL-6 was 7.24±0.92 ng/l, MMP-2 was 0.608±0.07 μg/l and TIMP-2 was 1739.31±254.39 pg/l. No significant difference (P>0.05) was identified between the model and middle-dose XMJ groups. The levels of ICAM-1, VCAM-1, IL-1 and IL-6 decreased, while the levels of MMP-2 and TIMP-2 increased. Lovastatin and zhibituo demonstrated marked suppressive effects on the H_2_O_2_-induced increase in ICAM-1, VCAM-1, IL-1 and IL-6 levels and on the decrease in MMP-2 and TIMP-2 levels, which were all statistically significant (P<0.05) when compared with the model group. Compared with the middle-dose XMJ group, the levels of supernatant cytokines in the lovastatin with zhibituo groups exhibited no statistically significant differences (P>0.05). The suppressive effects of low-dose XMJ on the increase in ICAM-1, VCAM-1, IL-1 and IL-6 levels and on the decrease in MMP-2 and TIMP-2 levels in the HUVEC injury model induced by H_2_O_2_ were significantly weaker when compared with the middle-dose XMJ group (P<0.05). However, the suppressive effects of high-dose XMJ were weaker than those of middle-dose XMJ; the reason is yet to be determined (Table II).

### Effect of XMJ on the level of NF-κB in the supernatant of HUVECs induced by H_2_O_2_

**Figure 4:**
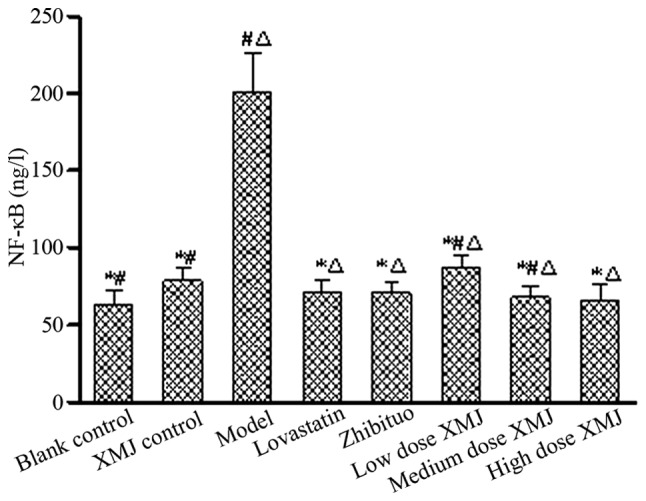
Effect of XMJ on the levels of NF-κB in the supernatant of HUVECs induced by H_2_O_2_. ^*^P<0.05, vs. model group; ^#^P<0.05, vs. high-dose XMJ group; ^△^P<0.05, vs. blank control group. XMJ, Xin Mai Jia; HUVECs, human umbilical vein endothelial cells; NF, nuclear factor; H_2_O_2_, hydrogen peroxide.

In the high-dose XMJ group, the concentration of NF-κB was 65.84±10.32 ng/l, whereas the level in the model group was 200.46±25.68 ng/l. Compared with the model group, the significant decrease in NF-κB concentration in the high-dose XMJ group was statistically significant (P<0.05). Lovastatin and zhibituo exhibited marked suppressive effects on the increase in supernatant NF-κB concentration in the HUVEC injury model induced by H_2_O_2_, which were statistically significant (P<0.05) when compared with the model group. However, when compared with the high-dose XMJ group, the differences in NF-κB concentration in the lovastatin and zhibituo groups were not statistically significant (P>0.05). The suppressive effects of the low- and middle-dose XMJ groups on the increase in NF-κB concentration in the HUVECs induced by H_2_O_2_ were significantly weaker when compared with the high-dose XMJ group (P<0.05), thus, the effects were dependent on the dose of XMJ (Fig. 4).

### Effect of XMJ on the level of NO in the supernatant of HUVECs induced by H_2_O_2_

**Figure 5:**
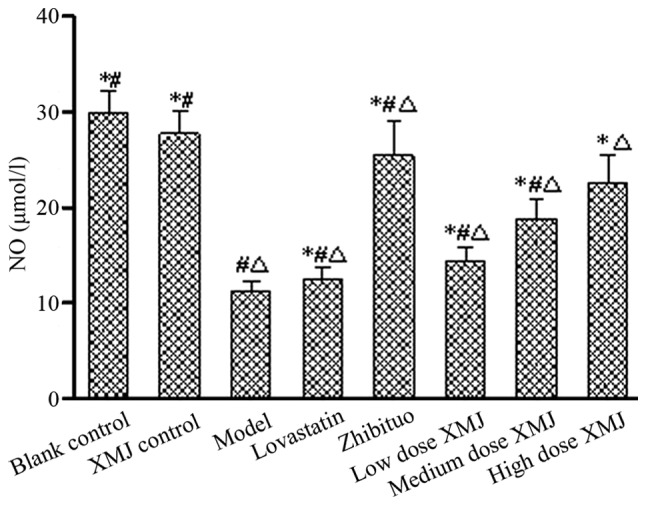
Effect of XMJ on the level of NO in the supernatant of HUVECs induced by H_2_O_2_. ^*^P<0.05, vs. model group; ^#^P<0.05, vs. high-dose XMJ group; ^△^P<0.05, vs. blank control group. XMJ, Xin Mai Jia; HUVECs, human umbilical vein endothelial cells; NO, nitric oxide; H_2_O_2_, hydrogen peroxide.

In the high-dose XMJ group, the concentration of NO was 22.58±2.58 μmol/l, while in the model group, the concentration was 11.21±1.11 μmol/l. The increase in NO concentration in the high-dose XMJ group exhibited a statistically significant difference (P<0.05) when compared with the model group. Lovastatin and zhibituo exhibited marked suppressive effects on the decrease in NO concentration in HUVECs induced by H_2_O_2_, which revealed statistically significant differences (P<0.05) when compared with the model group. However, the effect of the high-dose XMJ was more significant than that of lovastatin and zhibituo. The suppressive effects of low- and middle-dose XMJ on the H_2_O_2_-induced decrease in NO levels in the HUVECs were significantly weaker than that of high-dose XMJ (P<0.05), indicating that the effects were dependent on the dose of XMJ (Fig. 5).

### Detection of eNOS concentration using western blot analysis

**Figure 6:**
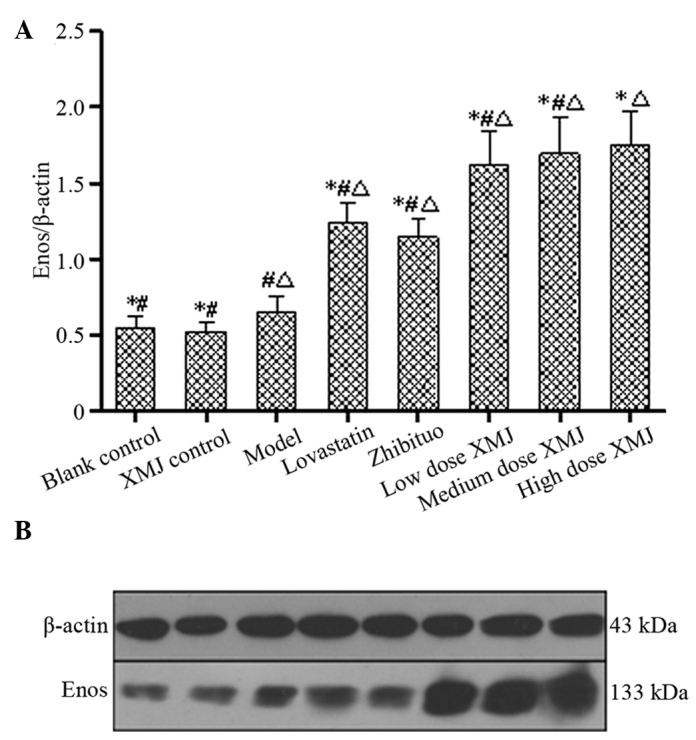
(A) Densitometric analysis of (B) western blot analysis showing the protein expression levels of eNOS in HUVECs.^*^P<0.05, vs. model group; ^#^P<0.05, vs. high-dose XMJ group; ^△^P<0.05, vs. blank control group. XMJ, Xin Mai Jia; HUVECs, human umbilical vein endothelial cells; eNOS, endothelial nitric oxide synthase.

The concentration of eNOS/β-actin was 1.75±0.22 in the high-dose XMJ group and 0.65±0.11 in the model group. The significant increase in eNOS/β-actin levels observed in the high-dose XMJ group was statistically significant (P<0.05) when compared with the model group. Lovastatin and zhibituo exhibited marked suppressive effects on the decrease in eNOS concentration in the HUVECs induced by H_2_O_2_, which was statistically significant (P<0.05) when compared with the model group. The suppressive effect of high-dose XMJ on the decrease in eNOS content was more significant than those of lovastatin and zhibituo (P<0.05). In addition, the levels of eNOS/β-actin in the low- and middle-dose XMJ groups were significantly weaker than that of the high-dose XMJ group (P<0.05), indicating that the effects were dependent on XMJ dosage (Fig. 6).

### Determination of eNOS protein concentration using immunohistochemistry

**Figure 7:**
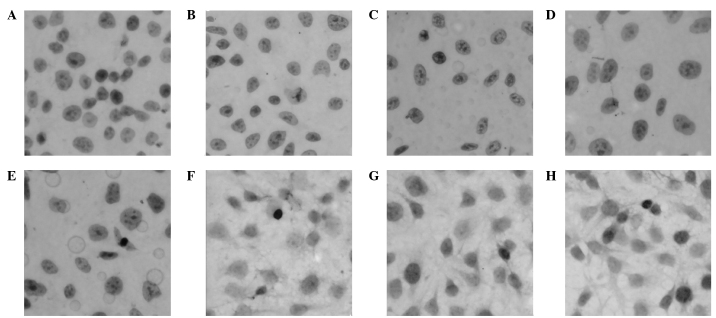
Immunohistochemical analysis (immunohistochemistry, Mayer’s hematoxylin counterstain) was used to detect the eNOS protein content in HUVECs (magnification, ×400) in the (A) blank control, (B) XMJ control, (C) model, (D) lovastatin, (E) zhibituo, (F) low-dose XMJ, (G) medium-dose XMJ and (H) high-dose XMJ groups. XMJ, Xin Mai Jia; HUVECs, human umbilical vein endothelial cells; eNOS, endothelial nitric oxide synthase.

In the high-dose XMJ group, the degree of eNOS staining saturation was 7.23±0.29%, the chromaticity was 42.55±3.27%, the grayscale value was 254.33±11.39%, the red value was 153.98±12.97%, the green value was 178.98±15.23% and the blue value was 165.38±11.27%. In the model group, the degree of eNOS staining saturation was 16.55±0.67%, the chromaticity was 33.78±2.17, the grayscale value was 189.46±12.54, the red value was 136.59±11.22, the green value was 154.78±13.24 and the blue value was 144.29±12.67. A significant difference (P<0.05) was observed in the eNOS coloration index between the high-dose XMJ and model groups. Lovastatin and zhibituo exhibited significant effects on the eNOS coloration index of the HUVEC model induced by H_2_O_2_, which were statistically significant (P<0.05) when compared with the model group. The eNOS staining indicators in the high-dose XMJ group were greater than those in the lovastatin and zhibituo groups (P<0.05). The effect of low- and middle-dose XMJ on the eNOS coloration index in the HUVECs induced by H_2_O_2_ were markedly weaker than that of the high-dose XMJ group, indicating XMJ dose dependence (Fig. 7).

### Determination of eNOS content using immunofluorescence

**Figure 8:**
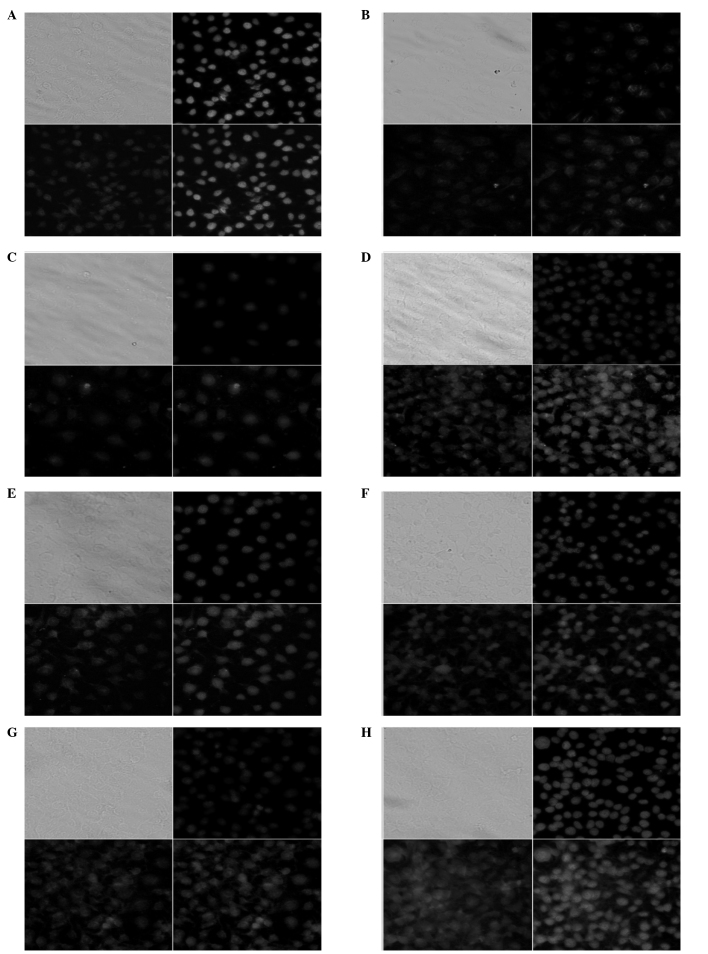
Immunofluorescence analysis was used to detect the levels of eNOS in HUVECs (magnification, ×400) in the (A) blank control, (B) XMJ control, (C) model, (D) lovastatin, (E) zhibituo, (F) low-dose XMJ, (G) medium-dose XMJ and (H) high-dose XMJ groups. XMJ, Xin Mai Jia; HUVECs, human umbilical vein endothelial cells; eNOS, endothelial nitric oxide synthase.

Confocal fluorescence tomography was performed on immunofluorescence cells with a laser scanning confocal microscope. The 32 facets of each cell were scanned and the fluorescence intensity within the cells was detected using fluorescence quantitative analysis software. The eNOS protein was predominantly expressed in the cytoplasm of the HUVECs. The positive signals presented yellowish-green spotlight, with diffused distribution. The fluorescence intensity values of the eNOS protein were 178.33±11.26 in the high-dose XMJ group and 65.27±4.66 in the model group, which exhibited a statistically significant difference (P<0.05). Lovastatin and zhibituo significantly increased (P<0.05) the fluorescence intensity of the eNOS protein in the HUVECs induced by H_2_O_2_. However, the fluorescence intensity of the eNOS protein in the high-dose XMJ group was greater than those in the lovastatin and zhibituo groups (P<0.05) The fluorescence intensities of the eNOS protein in the HUVECs treated with low- and middle-dose XMJ were significantly weaker than that of high-dose XMJ (P<0.05), indicating XMJ dose dependence (Fig. 8).

### Detection of eNOS gene expression levels using fluorescence qPCR

**Figure 9:**
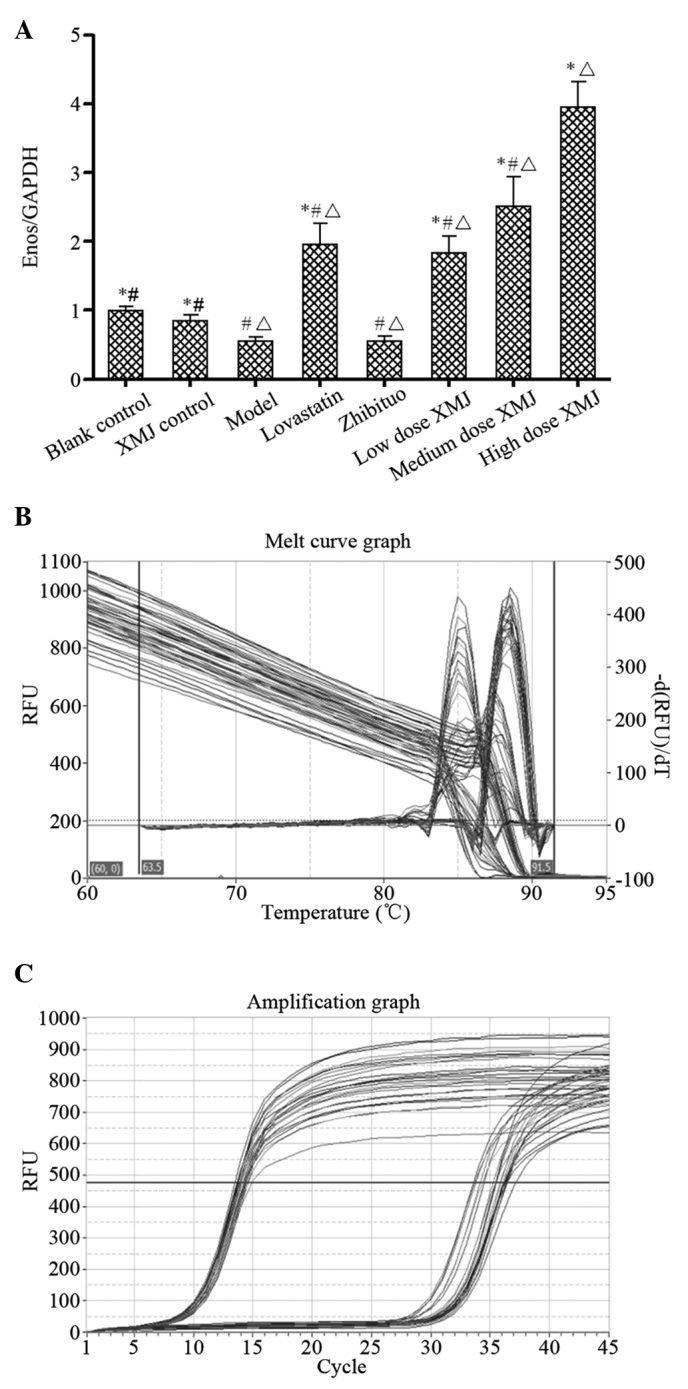
Fluorescence qPCR was used to detect the gene expression levels of eNOS in HUVECs. (A) Gene expression levels; (B) Melt curve; (C) amplification curve.^*^P<0.05, vs. model group; ^#^P<0.05, vs. high-dose XMJ group; ^△^P<0.05, vs. blank control group. XMJ, Xin Mai Jia; HUVECs, human umbilical vein endothelial cells; eNOS, endothelial nitric oxide synthase; qPCR, quantitative polymerase chain reaction.

Fluorescence intensity values of eNOS gene expression were 3.96±0.36 in the high-dose XMJ group and 0.55±0.77 in the model group; the results exhibited a statistically significant difference (P<0.05). Lovastatin and zhibituo significantly increased (P<0.05) the fluorescence intensity of eNOS gene expression in the HUVECs induced by H_2_O_2_ when compared with the model group. The fluorescence intensity of eNOS gene expression in the high-dose XMJ group was greater than those of the lovastatin and zhibituo groups (P<0.05). The fluorescence intensities of eNOS gene expression in the low- and middle-dose XMJ groups were significantly weaker than that of the high-dose XMJ group (P<0.05), thus, the effects were dependent on XMJ dosage (Fig. 9).

## Discussion

NO in the body results from the activation of the N-methyl-D-aspartate receptor and the catalysis of NOS. NOS widely exists in the nervous system, internally and externally. NOS isoenzymes are classified into three subtypes: Neuronal NOS (nNOS), eNOS and immune-NOS. nNOS and eNOS have Ca2+-dependent structural expressions, which exist in normal physiological state (15). NF-κB, an important transcription factor, regulates the expression of various genes involved in inflammation and immune processes, and is closely associated with several important pathophysiologies, including cell proliferation, transformation and apoptosis (16). In the development of AS, the expression of NF-κB, induced by a variety of pathogenic factors, increases, stimulating the increased secretion of IL-1, IL-6, ICAM-1, VCAM-1 and other inflammatory cytokines. In addition, NF-κB inhibits eNOS expression, reduces NO release and decreases normal vasomotion function. As a secondary intracellular messenger in HUVECs, NO increases the concentration of cGMP via the cGMP pathway. This phenomenon can influence ion channels or phosphodiesterase activity, activate cGMP-dependent protein kinase, activate cycloxygenase, protein kinase C and iron regulatory proteins, stimulate the expression of the early gene response or inhibit NF-κB and other non-cGMP pathways.

The majority of studies show that XMJ exhibits a marked protective effect on HUVEC injury induced by H_2_O_2_. This protective effect is dependent on the dose of XMJ (17,18). However, by studying the effect that XMJ has on the level of supernatant cytokines in HUVECs induced by H_2_O_2_, the protective effects of low- and middle-dose XMJ were shown to be dependent on XMJ dosage to a certain extent, whereas the suppressive effects of high-dose XMJ were weaker than those of middle-dose XMJ. In the high-dose XMJ group, cytokine levels were as follows: ICAM-1, 39.29±4.57 ng/l; VCAM-1, 43.38±2.39 μg/l; IL-1, 16.07±2.55 ng/l; IL-6, 7.21±0.98 ng/l; MMP-2, 0.643±0.07 μg/l; and TIMP-2, 1579.37±123.47 pg/l. This variation may possibly be attributed to the disproportion between the secretion and expression levels of the cytokines within the HUVEC supernatant, however, the specific reasons require further investigation.

MMP-2 belongs to the MMP family, which requires Ca^2+^, Zn^2+^ and other metal ions as cofactors for protein hydrolysis. MMP-2 consists of five functional domains. Firstly, a hydrophobic signaling peptide sequence. Secondly, the pre-peptide region, which functions in maintaining the stability of the proenzyme, as the MMP-2 zymogen is activated when the region is cut off by an exogenous enzyme. Thirdly, the catalytic activity area, which is a Zn^2+^ binding site that plays a crucial role in enzyme catalysis. Fourthly, the hinge region that contains abundant proline residues. Finally, there is the carboxyl terminal region, which is associated with the specificity of the enzyme substrate. The MMP-2 gene, which consists of 13 exons and 12 introns, is located on the 16q21 human chromosome. The total length of the structural gene is 27 kb, which differs from other MMPs due to the MMP-2 gene 5′ flanking sequence that contains two GC boxes instead of two TATA sequences, which promotes the subregion (19). MMP-2 can decompose the stromal component among the cells and type IV collagen, which is the main component of the basement membrane (20–22). Previous studies have reported that the expression of MMP-2 increases during early AS pathogenesis. However, following drug intervention, MMP-2 expression decreases, which confirmed the increase in MMP-2 to be an iconic indicator of AS (23–29). However, experimental data have produced contradictory results. In the present study, the level of MMP-2 in the model group was 0.608±0.07 μg/l, which was a marked decrease when compared with the blank control group, whereas the level of MMP-2 in the drug groups increased significantly. This difference may be associated with the secretion and disproportionate expression levels of cytokines in the supernatant fluid of the HUVECs, however, if XMJ is able to increase the levels of MMP-2, then the degradation of matrix proteins increases. This difference may be relevant with the ablation of the AS plaque. In the present study, the level of TIMP-2 in the drug groups increased, which may be attributable to a feedback mechanism from the body.

In order to determine whether XMJ itself exhibits harmful effects on the HUVEC model, which may have significantly affected the experimental data, a control group of XMJ drug media was used in the experiment. The results demonstrated that XMJ itself presents no harm to the HUVECs and does not affect the experimental results. Lovastatin and zhibituo are chemical drugs and TCM commonly used for the treatment of AS in clinical practice. Lovastatin and zhibituo were selected as the control drugs to ascertain the degree of the protective effect of XMJ on HUVEC injury induced by H_2_O_2_. The majority of the results indicated that the protective effects of XMJ on HUVEC injury induced by H_2_O_2_ were greater than those of lovastatin and zhibituo. However, the differences in the levels of SOD, MDA and NF-κB in the HUVEC supernatant among the high-dose XMJ, lovastatin and zhibituo groups presented no statistical significance (P>0.05). In addition, the difference in the levels of cytokines in the HUVEC supernatant among the middle-dose XMJ, lovastatin and zhibituo groups was not statistically significant (P>0.05). We hypothesize that these observations may have resulted from the dilution of cytokines in the HUVEC supernatant fluid or may be associated with the level of secretion. In conclusion, Xin Mai Jia prevents atherosclerosis and is superior to routine anti-atherosclerosis drugs such as lovastatin and Zhibituo.

## Figures and Tables

**Table I:** Protective effects of XMJ on the H_2_O_2_-induced decrease in the permeability of the HUVEC monolayer (n=6; mean ± SE).

Group	Jv (μl/min/cm^2^)	Kf (μl/min/cm^2^/Kpa)	σ
Blank control	27.26±4.63^a^,^b^	11.34±2.87^a^,^b^	0.54±0.07^a^,^b^
XMJ control	28.43±5.35^a^,^b^	11.54±2.89^a^,^b^	0.55±0.07^a^,^b^
Model	44.47±8.56^b^,^c^	17.66±3.43^b^,^c^	0.29±0.03^b^,^c^
Lovastatin	33.45±6.49^a^,^b^,^c^	14.46±2.98^a^,^b^,^c^	0.38±0.07^a^,^b^,^c^
Zhibituo	35.32±5.12^a^,^b^,^c^	14.64±2.39^a^,^b^,^c^	0.43±0.06^a^,^b^,^c^
Low-dose XMJ	34.34±5.43^a^,^b^,^c^	14.54±2.32^a^,^b^,^c^	0.41±0.08^a^,^b^,^c^
Medium-dose XMJ	30.12±5.67^a^,^b^,^c^	12.28±2.13^a^,^b^,^c^	0.46±0.08^a^,^b^,^c^
High-dose XMJ	29.43±7.53^a^,^c^	12.43±2.24^a^,^c^	0.59±0.08^a^,^c^

a P<0.05, vs. model group;

b P<0.05, vs. high-dose XMJ group;

c P<0.05, vs. blank control group.

XMJ, Xin Mai Jia; HUVEC, human umbilical vein endothelial cell; SE, standard error; Kf, endothelial monolayer filtration coefficient; σ, osmotic reflection coefficient; Jv, total water flux; H_2_O_2_, hydrogen peroxide.

**Table II:** Effect of XMJ on the levels of supernatant cytokines in HUVECs induced by H_2_O_2_ (n=6; mean ± SE).

Group	ICAM-1 (ng/l)	VCAM-1 (μg/l)	IL-1 (ng/l)	IL-6 (ng/l)	MMP-2 (μg/l)	TIMP-2 (pg/l)
Blank control	28.91±3.65^a^,^b^	36.49±1.22^a^,^b^	14.23±1.25^a^,^b^	5.93±0.45^a^,^b^	0.622±0.09^a^,^b^	1925.88±236.12^a^,^b^
XMJ control	36.33±4.39^a^,^b^	38.78±2.39^a^,^b^	13.93±1.36^a^,^b^	6.51±0.65^a^,^b^	0.647±0.07^a^,^b^	2084.74±268.99^a^,^b^
Model	71.18±6.67^b^,^c^	44.81±2.09^b^,^c^	18.34±2.14^b^,^c^	7.24±0.92^b^,^c^	0.608±0.07^b^,^c^	1739.31±254.39^b^,^c^
Lovastatin	38.55±4.16^a^,^c^	35.84±1.96^a^,^c^	14.63±1.57^a^,^c^	6.05±0.78^a^,^c^	0.683±0.08^a^,^c^	1742.06±156.36^a^,^c^
Zhibituo	34.10±4.12^a^,^c^	36.49±2.68^a^,^c^	14.79±1.69^a^,^c^	6.19±0.65^a^,^c^	0.622±0.07^a^,^c^	1475.53±124.82^a^,^c^
Low-dose XMJ	62.78±5.47^a^,^b^,^c^	38.94±2.47^a^,^b^,^c^	15.49±2.31^a^,^b^,^c^	6.48±0.77^a^,^b^,^c^	0.612±0.06^a^,^b^,^c^	2137.27±213.29^a^,^b^,^c^
Medium-dose XMJ	36.33±7.32^a^,^c^	36.66±1.58^a^,^c^	14.75±2.04^a^,^c^	6.05±0.84^a^,^c^	0.671±0.07^a^,^c^	2605.99±222.17^a^,^c^
High-dose XMJ	39.29±4.57^a^,^b^,^c^	43.38±2.39^a^,^b^,^c^	16.07±2.55^a^,^b^,^c^	7.21±0.98^a^,^b^,^c^	0.643±0.07^a^,^b^,^c^	1579.37±123.47^a^,^b^,^c^

a P<0.05, vs. model group;

b P<0.05, vs. medium-dose XMJ group;

c P<0.05, vs. blank control group.

XMJ, Xin Mai Jia; HUVECs, human umbilical vein endothelial cells; SE, standard error; ICAM, intercellular adhesion molecule; VCAM, vascular cell adhesion molecule; IL, interleukin, MMP, matrix metalloproteinase; TIMP, tissue inhibitor of metalloproteinase; H_2_O_2_, hydrogen peroxide.
